# Retraction: Investigating capital flight in South Asian countries: The dual influence of terrorism and corruption

**DOI:** 10.1371/journal.pone.0331595

**Published:** 2025-09-04

**Authors:** The PLOS One Editors

The *PLOS One* Editors retract this article [1] because it was identified as one of a series of submissions for which we have concerns about potential manipulation of the publication process, peer review integrity, and authorship. These concerns call into question the validity and provenance of the reported results. We regret that the issues were not identified prior to the article’s publication.

FK did not agree with the retraction. KA, WQ, and MA either did not respond directly or could not be reached.
